# A new type of C^+^⋯H^δ−^(C=) bond in adducts of vinyl carbocations with alkenes

**DOI:** 10.1038/s41598-024-58109-4

**Published:** 2024-04-10

**Authors:** Evgenii S. Stoyanov, Irina Yu. Bagryanskaya, Irina V. Stoyanova

**Affiliations:** Vorozhtsov Institute of Organic Chemistry, Siberian Branch of Russian Academy of Sciences, Novosibirsk, 630090 Russia

**Keywords:** Chemistry, Materials science

## Abstract

By X-ray diffraction analysis and IR spectroscopy, it was established here that vinyl carbocations C_3_H_5_^+^/C_4_H_7_^+^ with carborane counterion CHB_11_Cl_11_^−^ form stable monosolvates C_3_H_5_^+^⋅C_3_H_6_/C_4_H_7_^+^⋅C_4_H_8_ with molecules of alkenes C_3_H_6_/C_4_H_8_. They contain molecular group =C^+^⋯H^δ−^–C^δ+^= with a new type of bond formed by the H atom of the H–C= group of the alkene with the C atom of the C^+^=C group of the carbocation. The short C^+^----C^δ+^ distance, equal to 2.44 Å, is typical of that of X----X in proton disolvates (L_2_H^+^) with an quasi-symmetrical X–H^+^⋯X moiety (where X = O or N) of basic molecule L. The nature of the discovered bond differs from that of the classic H–bond by an distribution of electron density: the electron–excessive H^δ−^ atom from the (=)C–H group of the alkene is attached to the C^+^ atom of the carbocation, on which the positive charge is predominantly concentrated. Therefore, it can be called an inverse hydrogen bond.

## Introduction

Vinyl carbocations are known as an important class of reactive intermediates for organic synthesis^1^ and have been extensively investigated via theoretical calculations and experimental methods^2–4^. From studies on solvolysis reactions, it has been concluded that they are highly reactive and therefore difficult to study^5–7^. Nevertheless, Mayer and coworkers have shown that their expected reactivity is exaggerated^8^. Indeed, the benzyl carbocation acts as a protonating agent converting into a carbene molecule, the high reactivity of which can be attributed to the carbocation^9^. Due to the expected instability of vinyl carbocations not stabilized by electron-donating substituents (e.g., alkyl and aryl groups or heteroatoms), no active steps have been taken to isolate them in the form of pure salts. Accordingly, the formation of C_3_H_5_^+^ has been proven in a cryogenic superacid matrix (170 K) by IR spectroscopy^10,11^. On the other hand, with increasing temperature, the IR spectrum of the sample changes, from which it has been concluded that nonstabilized alkene carbocations are stable only at temperatures below − 100 °C. Numerous attempts have been made to study the C_3_H_5_^+^ cation by NMR spectroscopy in liquid superacids at low temperature, and they have failed^12^. It has been believed that research on vinyl cation properties in solution is complicated by very short life-times in most solvents. Therefore, experimental studies on the simplest cations C_2_H_3_^+^ and C_3_H_5_^+^ have been conducted only in vacuum by mass-selected IR spectroscopy^13,14^. Nevertheless, solid salts of vinyl cations C_3_H_5_^+^ and C_4_H_7_^+^ with carborane anions CHB_11_Cl_11_^−^ were recently obtained, and the structure of the cations was determined by X–ray diffraction analysis^15–17^. Salts of methyl-propargyl cation C_4_H_5_^+^ and chlorinated vinyl cations were also obtained, and their structure was determined by NMR^18^, X-ray, and IR spectroscopy^17,19^. It turned out that salts of vinyl cations with carborane counterions are stable at least up to 150 °C^15–18^. In solutions in common organochlorine solvents, they appear to be stabler than salts of alkyl carbocations and can be stored for a long time without decomposition under ambient conditions^20^. This state of affairs has opened up the possibility of studying the interaction of vinyl cations with nucleophile molecules in solutions in solvents common in chemical practice. The first study on the interaction of vinyl carbocations C_3_H_5_^+^ and i-C_4_H_7_^+^ with water, alcohol, and acetone molecules was published in Ref.^21^. Thus, research in the past 2–3 years shows that the properties of vinyl cations are very different from those predicted on the basis of earlier studies.

In the present work, we continue the investigation into the interaction of vinyl carbocations C_3_H_5_^+^ and *i*-C_4_H_7_^+^ with nucleophiles, such as alkene molecules, C_3_H_6_, and *i*-C_4_H_8_, respectively, by IR spectroscopy and X-ray analysis. As a counterion, undecachlorocarborane anion CHB_11_Cl_11_^−^ was chosen (Fig. S1 in Supporting Information [SI]). Its extreme stability and very low basicity promote the formation of stable salts with highly reactive cations^22^.

## Results

**Figure 1:**
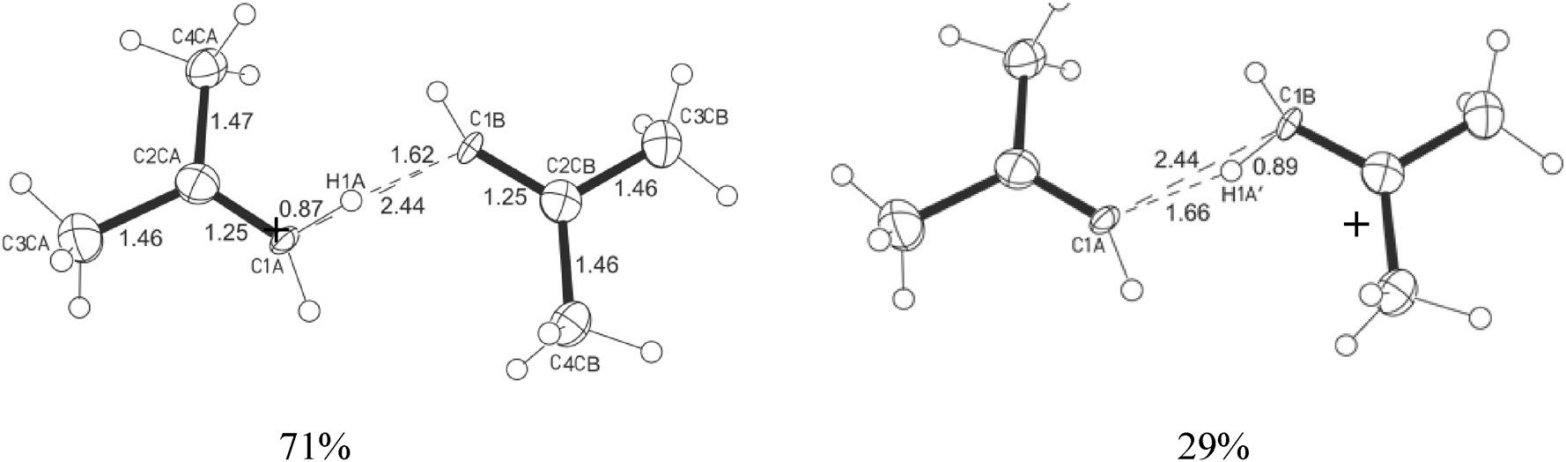
Structures of cationic adduct **I** with two positions of the bridging H atom: (left) with 71% occupancy and (right) with 29% occupancy. The bend C–H⋯C is 159.94° and 158.68°, respectively; the H⋯C distance is 1.597 Å for both.

During the growing of crystals of C_3_H_5_^+^ and C_4_H_7_^+^ cations’ salts with carborane anion CHB_11_Cl_11_^−^ (hereafter abbreviated as {Cl_11_^−^}) from their solutions in dichloromethane (DCM), the surface of the crystals was found to be always contaminated with a film of a viscous phase. IR spectra of this phase contain absorption bands of C=C vibrations of vinyl cations: a known one at 1490 cm^−1^ (Refs.^15–17^) and one or two new bands with the most intense one at 1536 cm^−1^, which belong to byproducts. To identify the main byproduct of the viscous phase, we washed contaminated crystals of the salt of the chain butylene cation (CH_3_–C^+^=CH–CH_3_){Cl_11_^−^} with a few drops of DCM (their preparation from a solution in DCM and their investigation are described in Ref.^17^). The washing solution was left to recrystallize. After 4 days, a few small crystals appeared. X-ray diffraction analysis of the first selected crystal showed that it is a known salt, C_4_H_7_^+^{Cl_11_^−^}, of the chain butylene cation^17^ with slightly disordered structure. The second, larger one, yielded no diffraction pattern, that is, it has glassy structure. The third small crystal showed a diffraction pattern, and after long accumulation, its structure was determined. It is a salt of the isobutylene cation solvated by the isobutylene molecule, C_4_H_7_^+^⋅C_4_H_8_, via the H atom of its CH_2_= group (Fig. 1). This H atom is disordered at two positions: at the C1A atom (H1A) and at the C1B atom (H1A’), as determined from a Fourier difference map (X-ray data, atomic parameters, and their designations are given in Tables S1 and S2 in SI). Refinement of the occupancy of two positions of the H1A atom indicated that near the C1A atom, it is 71%, and near the C1B atom, 29%. Thus, we see averaged geometry across the neutral molecule and cation, and this arrangement leads to identical positions of the carbon atoms in both entities. Positions of the hydrogen atoms for CH_3_-groups were calculated with the riding model. Hereinafter, this cationic adduct will be denoted as **I**, and its salt as salt **I**.

The IR spectrum of exactly a single crystal of salt **I**, which was subjected to X-ray diffraction analysis, could not be recorded because of its small size. We were able to record only a weak low-quality attenuated total reflectance (ATR) IR spectrum of the obtained set of tiny crystals.

**Figure 2:**
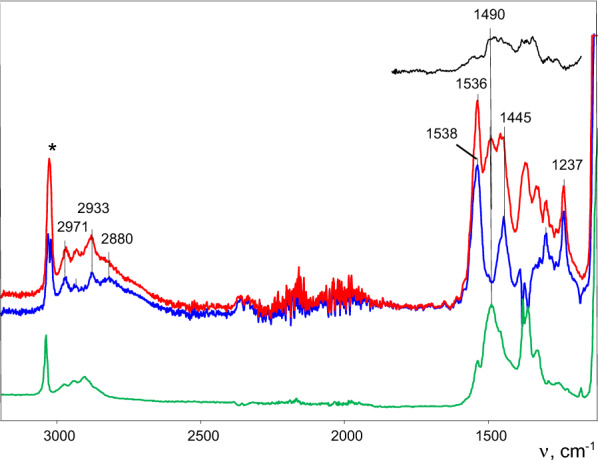
ATR-IR spectra of mixtures of a small amount of crystals, mainly salts C_4_H_7_^+^{Cl_11_^−^} and to a lesser extent salts C_4_H_7_^+^⋅C_4_H_8_{Cl_11_^−^} (black); solid grains of salt C_4_H_7_^+^⋅C_4_H_8_{Cl_11_^−^}, which did not show X-ray diffraction patterns (red), and crystals of the C_4_H_7_^+^{Cl_11_^−^} salt studied in Ref.^15^ (green). The result of subtraction of the green spectrum from the red one is blue. The band marked with * belongs to the CH stretch vibration of the {Cl_11_^−^} anion.

It contained a known νC=C band at 1490 cm^−1^ of the C_4_H_7_^+^ cation of the crystalline salt and a new one, of medium intensity, at 1536 cm^−1^, which is typical for spectra of the wax phase (Fig. 2, black). Therefore, the resulting set of crystals consists predominantly of salt C_4_H_7_^+^{Cl_11_^−^}. The band at 1536 cm^−1^ is well pronounced as a strong one in the IR spectrum of the solid grains obtained from a solution of pure salt C_4_H_7_^+^⋅C_4_H_8_{Cl_11_^−^} in DCM (Fig. 2, red), which is discussed below. Nonetheless, these grains did not give X-ray diffraction patterns. We assumed that the spectrum with band 1536 cm^−1^ belongs to the salt of adduct** I**. It also contains a very broad absorption pattern of the H_3_O^+^ cation at ~ 2900 cm^−1^^23^, whose intensity seems to depend on that of the band at 1536 cm^−1^. Apparently, the emergence of an alkene required for the formation of adduct **I** depends on the presence of traces of water, and this issue will be discussed below.

**Figure 3:**
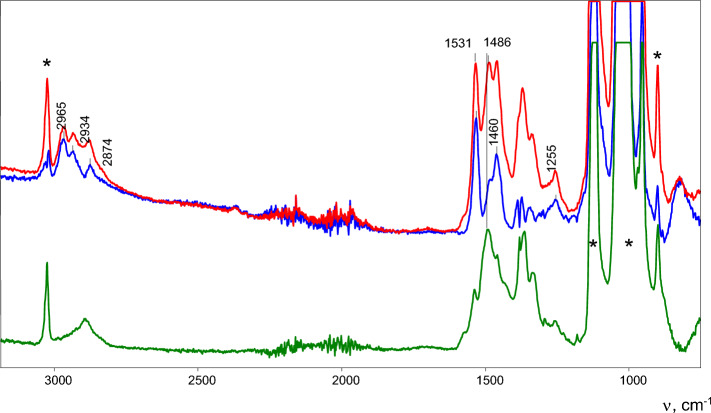
ATR-IR spectra of solid grains of salt C_3_H_5_^+^⋅C_3_H_6_{Cl_11_^−^} (red), which do not show X-ray diffraction patterns, and crystals of salt C_3_H_5_^+^{Cl_11_^−^}, researched in Ref.^16^ (green). The result of subtraction of the green spectrum from the red one is blue. The intense bands marked with an asterisk belong to the {Cl_11_^−^} anion.

The salt of a similar solvate of the propylene cation with a propylene molecule was obtained in pure form by wetting a powder of acid H{Cl_11_} with drops of 1-chloropropane until a viscous phase arose. It was dissolved in a small volume of DCM. Adding a few drops of hexane to it led to precipitation of the solid phase. Its chemical analysis revealed that it is a carborane salt with an organic cation of composition C_6.25_H_11.25_ (Table S3 in SI), which corresponds to the closest formulae C_6_H_11_. Experimental error of the determination of this composition may be relatively large, but taking into account that the starting reagent is a C_3_-hydrocarbon, it favors the formation of adduct C_3_H_5_^+^⋅C_3_H_6_. (This is confirmed by the close match between its IR spectrum and that of cation **I**, see Fig. 3, red, and Fig. 2, red.) Hereafter, we will denote this cation as **II**.

Attempts to grow crystals from solutions of salt **II** in DCM in order to analyze them by X-ray diffraction were unsuccessful: the precipitated grains looked like crystals but did not yield a diffraction pattern. Its IR spectrum, shown in Fig. 3 (red), coincides with that of the viscous phase of salt **II** and its very similar to the spectrum of salt **I** (Fig. 2, red), indicating the similarity of **I** and **II**.

IR spectra of cations **I** and **II** show two (actually identical) frequencies of C=C stretches at 1486 and 1531 cm^−1^. The first of them matches the C=C stretch in the IR spectra of individual cations C_3_H_5_^+^ and C_4_H_7_^+^ in their salts^15–17^ (Figs. 2 and 3, green). Therefore, the spectrum of salts of these cations with the band of the C=C stretch at 1486 cm^−1^ can be successfully subtracted from the spectrum of salts **I** or **II**, giving a difference spectrum with a band of the C=C stretch at 1531 cm^−1^ (and at 1460 cm^−1^ for bending vibrations of the CH_3_ groups), which obviously belongs to the cation-solvating propene or isobutene molecule (Figs. 2 and 3, blue). If the IR spectra of crystalline salt C_3_H_5_^+^{Cl_11_^−^} and the salt of the C_3_H_5_^+^⋅C_3_H_6_ cation (Fig. 3, green and red, respectively) are normalized to unit intensity of the {Cl_11_^−^} anion, then the intensity of the band of the C=C stretch is similar between the two spectra, and the spectrum of adduct **II** looks as if it contains an essentially unchanged spectrum of the free C_3_H_5_^+^ cation (Fig. S2 in SI). That is, the binding of alkene C_3_H_6_ to the C_3_H_5_^+^ cation has little effect on its spectrum.

## Discussion

The salt of C_3_H_5_^+^⋅C_3_H_6_ adduct **II** is formed in two stages. The first one is when a very small amount of 1-Cl-propane is added to a H{Cl_11_} powder, so that the phase remains solid. This is a known method for obtaining a salt of the C_3_H_7_^+^ alkane cation via the reaction^24^

1 $$ {\text{H}}\left\{ {{\text{Cl}}_{{{11}}} } \right\} + {\text{C}}_{{3}} {\text{H}}_{{7}} {\text{Cl}} \to {\text{C}}_{{3}} {\text{H}}_{{7}}^{ + } \{ {\text{Cl}}_{{{11}}}^{ - } \} + {\text{HCl}}. $$

The next step is to add some more 1-chloropropane to obtain a viscous liquid phase in which alkane carbocations have been found to spontaneously convert to vinyl carbocations^20^. In our case, this is the transition C_3_H_7_^+^ → C_3_H_5_^+^ + H_2_. An available excess of 1-chloropropane molecules in a strongly acidic medium decomposes (with a release of HCl) into propylene, which attaches to the vinyl cation giving rise to adduct **II**:

2 $$ {\text{C}}_{3} {\text{H}}_{5}^{ + } + {\text{C}}_{3} {\text{H}}_{7} {\text{Cl}} \to {\text{C}}_{3} {\text{H}}_{5}^{ + } \cdot {\text{C}}_{3} {\text{H}}_{6} + {\text{HCl}}. $$

The released gaseous HCl produced bubbles in the viscous phase and was identified by IR spectroscopy.

Reaction (2) proceeds with the decomposition of 1-chloropropane to HCl and propene; however this decomposition for neat molecules of 1-chloropropane is endothermic. The driving force behind this process is probably the formation of stable compound C_3_H_5_^+^⋅C_3_H_6_. Reactions (1 and 2) have been studied for chloroethane^25^ when the second stage stops during the formation of a salt of the chloronium cation, which decomposes—with an increase in temperature to 150 °C—to the *i*-Bu^+^ cation:

$$ {\text{C}}_{{2}} {\text{H}}_{{5}} \left\{ {{\text{Cl}}_{{{11}}} } \right\} + {\text{C}}_{{2}} {\text{H}}_{{5}} {\text{Cl}} \to \left( {{\text{C}}_{{2}} {\text{H}}_{{5}} {\text{-Cl}}{\text{-C}}_{{2}} {\text{H}}_{{5}} } \right)^{ + } \{ {\text{Cl}}_{{{11}}}^{ - } \} \to {\text{i}}{\text{-C}}_{{4}} {\text{H}}_{{9}}^{ + } \{ {\text{Cl}}_{{{11}}}^{ - } \} + {\text{HCl}}. $$

Next, it reacts with an excess of C_2_H_5_Cl thereby generating oligomeric solvates^25,26^:

$$ i{\text{-C}}_{{4}} {\text{H}}_{{9}}^{ + } \left\{ {{\text{Cl}}_{{{11}}}^{ - } } \right\} + {\text{nC}}_{{2}} {\text{H}}_{{5}} {\text{Cl}} \to i{\text{-C}}_{{4}} {\text{H}}_{{9}}^{ + } \left( {{\text{C}}_{{2}} {\text{H}}_{{5}} } \right)_{{\text{n}}} \left\{ {{\text{Cl}}_{{{11}}}^{ - } } \right\} + {\text{nHCl}}, $$

where n = 0 to 4. Chloropropane reacts similarly to form the chloronium cation^26^.

$$ {\text{C}}_{{3}} {\text{H}}_{{7}}^{ + } \{ {\text{Cl}}_{{{11}}}^{ - } \} + {\text{C}}_{{3}} {\text{H}}_{{7}} {\text{Cl}} \to \left( {{\text{C}}_{{3}} {\text{H}}_{{7}}{\text{-Cl}}{\text{-C}}_{{3}} {\text{H}}_{{7}} } \right)^{ + } \{ {\text{Cl}}_{{{11}}}^{ - } \} , $$

which, when the temperature rises to 100 °C, decomposes to C_6_H_15_^+^{Cl_11_^−^} with the release of HCl. The (C_3_H_7_–Cl–C_3_H_7_)^+^ cation in its salt with the {Cl_11_^−^} anion is unstable at room temperature and is in equilibrium with its decomposition products^26^. The IR spectrum of these products does not contain absorption bands of the C_6_H_15_^+^ and C_3_H_7_^+^ cations but shows the absorption pattern of the C_3_H_5_^+^⋅C_3_H_6_ cation, which we are studying. Therefore, at room temperature, the chloronium salt decomposes to the salt of the solvated propylene cation.

$$ \left( {{\text{C}}_{{3}} {\text{H}}_{{7}} {-}{\text{Cl}}{-}{\text{C}}_{{3}} {\text{H}}_{{7}} } \right)^{ + } \{ {\text{Cl}}_{{{11}}}^{ - } \} \to {\text{C}}_{{3}} {\text{H}}_{{5}}^{ + } \cdot {\text{C}}_{{3}} {\text{H}}_{{6}} \{ {\text{Cl}}_{{{11}}}^{ - } \} + {\text{HCl}} + {\text{H}}_{{2}} . $$

This reaction with the formation of C_3_H_5_^+^⋅C_3_H_6_{Cl_11_^−^} and HCl (it is necessary to detect the release of H_2_) is an experimentally established fact. Its explanation requires additional research.

The salt of adduct **I** can be prepared similarly to salt **II,** but it was impossible to obtain high-quality crystals from it. Crystals suitable for X-ray diffraction analysis were obtained as a byproduct formed in a DCM solution of the C_4_H_7_^+^{Cl_11_^−^} salt with the chain carbocation, when stored for several days. This happened because two hydrocarbon components—the iso-C_4_H_7_^+^ cation and iso-butylene, necessary for the formation of adduct **I**—form very slowly. Thus, the transition of chain cation CH_3_–C^+^=CH–CH_3_ to the iso-C_4_H_7_^+^ cation, according to quantum-chemical calculations, is unfavorable^15^, and equilibrium concentration of the iso-C_4_H_7_^+^ cation in solution is negligible. Most likely, both the iso-C_4_H_7_^+^ cation and isobutylene form from traces of chlorobutane isomers contained as impurities in the 1,4-dichlorobutane used to prepare vinyl cation salts. When the 1-chlorobutane impurity (and other isomers) reacts with acid H{Cl_11_}, a salt of the t-Bu^+^ cation is produced^27^ (a tertiary cationic center in carbocations arises from the well-known rearrangement of the initially formed primary carbocation into a stabler tertiary cation via rapid 1,2 shifts^28^), which in the liquid phase converted into the vinyl iso-C_4_H_7_^+^ cation within dozens of minutes^20^. The source of isobutylene may also be the admixture of t-Bu^+^ cations because its formation is accompanied by the formation of the H_3_O^+^ cation from traces of water molecules according to the equation.

$$ {\text{t-C}}_{{4}} {\text{H}}_{{9}}^{ + } + {\text{H}}_{{2}} {\text{O}} \to {\text{C}}_{{4}} {\text{H}}_{{8}} + {\text{H}}_{{3}} {\text{O}}^{ + } . $$

Because the content of water molecules in the reaction mixture is negligible and can increase very slowly over time (the solution is stored in a glove box), the formation of isobutylene molecules in the solution proceeds very slowly. Therefore, under these conditions, small crystals of salt **I,** having the high quality necessary for their X-ray diffraction analysis, grow for 4 or more days.

**Figure 4:**
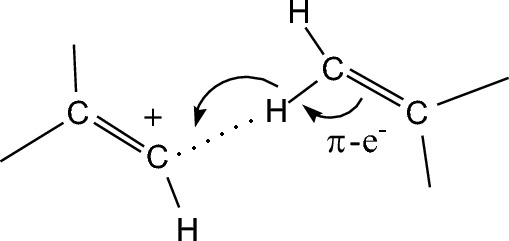
Schematic representation of the nature of the bond in adduct** I** formed by the supply of π-electron density from the C=C bond of an alkene to the positively charged site of the cation.

The structure of **I** is interesting in that the C1A–C1B distance between the C atoms of the cation and the alkene molecule is as short, 2.44 Å, as between the X atoms in the X–H^+^⋯X part of the molecule in proton disolvates L–H^+^⋯L (where L are base molecules with X atoms of O or N) with a strong (L–)H^+^⋯L hydrogen bond^29–35^. Adduct **I** is topologically similar to the currently unknown proton disolvate with the C–H⋯C^+^ moiety, whose nature is different from that of classic proton disolvates: the H atom interacts with the positively charged C^+^ atom by acting as an electron donor. In case of **I**, this can occur if under the influence of the positive charge of the cation, π-electron density of the C=C bond in the alkene is drawn to the σ C–H bond and next to the carbon atom of the C=C^+^ bond of the cation, as illustrated in Fig. 4.

This pattern of interactions is in agreement with the IR spectra. Decreasing in the π–C=C electron density of alkene molecules leads to a decrease in their C=C stretch frequency by 107–105 cm^−1^ as compared to free molecules (1645–1640 cm^−1^) but keeps it at 48–45 cm^−1^ higher relative to the vinyl cations (1490 cm^−1^). Accordingly, electron density transfer to the conjugated CH bond strengthens it (C–H shortens to 0.88 Å) and the CH stretching frequencies should increase (discussed below). Negative charge δ^−^ arising on the H atom gives this atom weak properties of a hydride with the ability to interact with the charged C^+^ atom of the cation.

The C^+^=C stretch frequency of the *i*-C_4_H_7_^+^/C_3_H_5_^+^ cation of adduct **I/II** almost matches that of the “free” cation (Fig. S2 in SI), which means that the π-electron density supplied from the alkene via the H atom to the H⋅⋅⋅C^+^(=C) bond actually does not reach the C^+^=C bond of the cation. That is, the nature of the H⋅⋅⋅C^+^(=C) bond is ionic without a noticeable covalent component.

Thus, the C^δ+^–H^δ−^···C^+^ moieties of **I** and **II**, while showing topological similarity with the X–H^+^···X moieties of proton disolvates, fundamentally differ from them by the inverse charge distribution. Therefore, their H^δ−^···C^+^ bond, in contrast to the classic H-bond can be called an inverse hydrogen bond. The term “inverse hydrogen bond” was suggested in a theoretical study that reflects this chemical situation^36^.

Despite the reversal of the charge distribution in moieties X–H^+^···X and C^δ+^–H^δ−^···C^+^, their geometric parameters determined by X-ray diffraction can serve as indicators of the strength of their H^+^···X and H^δ−^···C^+^ bonds. Firstly, it is usually accepted that the strength of interactions in X–H^+^⋯X moieties is characterized by the X⋯X distance and bond lengths H^+^⋯X and H⋯C^+^^31,33^. If the X⋯X distance is less than ~ 2.5 A˚ for O–H⋯O or 2.6–2.7 A˚ for N–H⋯N parts of the molecule, then this indicates the presence of a strong H-bond^31,34^. The C atoms in the C^δ+^–H^δ−^···C^+^ moiety have a larger radius (1.70 Å) than the N (1.55 Å) or O atoms (1.52 Å)^35^, and the sum of the van der Waals radii 2C (3.54 Å) is greater than 2N (3.27) and 2O (3.16 Å). Therefore, at the same bond strength, distance C⋯C in the C^δ+^–H^δ−^···C^+^ moiety will slightly exceed distance X…X in the X − H^+^⋯X moieties with X=O or N. Therefore, the C···C distance in C^δ+^–H^δ−^···C^+^ (2.44 Å) almost equals to ~ 2.5 A˚, meaning that this part of the molecule contains a sufficiently strong reverse H-bond.

The second piece of evidence proving the existence of an H-bond/reverse H-bond in the corresponding fragments and assessing their strength, is the lengths of the bonds X–H^+^ or C^δ+^–H^δ−^ and H^+^⋯X or H^δ−^⋯C^+^. In the X–H^+^⋯X parts of the molecule the X–H^+^ bond is elongated (it is weakened) and the length of H^+^···X is much less than the sum of van der Waals radii of H and X. In the C^δ+^–H^δ−^⋯C^+^ moiety, the transfer of π-electron density to the C^δ+^–H^δ−^ bond strengthened it and significantly shortened it to 0.88 Å as compared to the standard value of 0.98 Å. The second distance H^δ−^⋯C^+^ (1.597 Å) is much smaller than the sum of van der Waals radii of H and C (2.88 Å). According to Jeffrey’s classification^37^, for strong H-bonds, the H⋯X distance is 1.2–1.5 Å. Considering that the radius of the C atom is greater than that of the O or N atoms, the available evidence suggests that the strength of the reverse H-bond being discussed is comparable to that of a moderately strong classic H-bond.

Finally, the third indicator of bond strength in the X–H^+^···X part of the molecule is the X–H–X bending angle. For strong H-bonds, these angles are 170–180°; for moderately strong H-bonds, they are > 130°^37^. For the C^+^···H^δ−^ − C^δ+^ moiety, the angle is ~ 160°, which again corresponds to the borderline state between a medium and strong inverse H-bond.

An indirect confirmation of the strength of the inverse H-bond in **I** and **II** is the finding that these compounds turned out to be thermally stable at least up to 120–150 °C.

The C^+^···H^δ−^ − C^δ+^ moiety is asymmetric in accordance with the fact that the two positions of the bridging H-atom were determined from the Fourier difference map of X-ray analysis (Fig. 1). This asymmetry is also evidenced by asymmetric interaction of the C^+^···H^δ−^ − C^δ+^ with Cl atoms of the anions in the immediate environment (Fig. S3 in SI). The potential of bridge H is a double-well, and a quick H exchange can occur between the two minima:
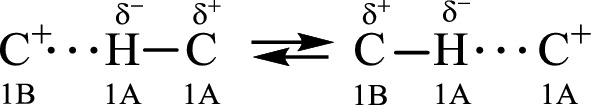


In the case of a classic H-bond of medium strength, as in H complexes of carboxylic acids^38^, a quick proton exchange manifests itself in the IR spectra as an intense and very broad absorption pattern of C–H^+^ stretch vibrations in the frequency range 2800–2500 cm^−1^. The absorption of moiety C^+^···H^δ−^ − C^δ+^ should manifest itself in the form of absorption of C–H^+^ stretch with an increased frequency (above 2900–2800 cm^−1^) and reduced intensity. If the potential barrier separating the two potential energy minima is low and the H^δ−^ exchange at the time scale of IR spectroscopy is quite fast, then the absorption of the C–H^+^ stretch will be greatly broadened. Given its reduced intensity, this phenomenon will make it difficult to detect. Probably for this reason, the absorption band of the C–H^+^ stretch could not be detectable in the IR spectra of **I** and **II**.

We attempted to characterize the structure of adduct **II** by quantum chemical calculations at the UB3LYP/6-311++G(d,p) level of theory with the Grimme dispersion correction. The obtained results are presented in SI. They showed that adducts with structures **I** and **II** are energetically unfavorable and should not exist. The use of other algorithms yielded the same result, which does not depend on taking into account the surrounding anions. Previously, we have also encountered difficulties with using a quantum chemical calculation in an experimental study on saturated and unsaturated non aromatic carbocations^15,16,24,27^. Problems associated with poor compatibility of quantum chemical calculations with experimental data on alkane and vinyl carbocations require additional investigation.

In conclusion, it can be said that the formation of stable (under ambient conditions) salts of vinyl cations solvated by alkenes, C_3_H_7_^+^⋅C_3_H_6_ and C_4_H_9_^+^⋅C_4_H_8_, with carborane counterion {Cl_11_^−^} was proven experimentally. In them, the interaction of a neutral alkene with a cation leads to the formation of a C^+^∙∙∙H^δ−^ − C^δ+^ moiety with a short C–C distance of a 2.44 Å, which is formed by a strong-to-medium inverse hydrogen bond similar in strength to classic H-bonds in quasi-symmetric moieties X…H^+^–X in proton disolvates L_2_H^+^, where L is a basic molecule.

Such a bond does not contradict one of the first definitions of hydrogen bonds given by Pimentel and McClellan in 1960: "they exist when 1) there is evidence of a bond, and 2) there is evidence that this bond specifically involves a hydrogen atom already bonded to another atom^39^. This definition makes it possible to disregard the condition that the H atom of the CH group must always be electron-deficient, although the fulfillment of this condition is necessary for hydrogen bonding of a C–H group, and no exceptions to this rule are known^40^. Later, in 1993, Steiner and Sanger refined the definition of a hydrogen bond: “any cohesive interaction X–H⋯Y where H carries a positive and Y a negative (partial or full) charge and the charge on X is more negative than on H”^41^. It is closest to the modern concept of the nature of hydrogen bonding^32,42^. The nature of the bond in compounds **I** and **II** does not fall under this definition, because the charge distribution in the C^+^∙∙∙H^δ−^–C^δ+^ moiety is reverse to that which forms a conventional H-bond. Therefore, in Ref.^36^, which theoretically examined the possibility of forming such a bond, a term was proposed for its name: an inverse hydrogen bond.

From the results of the present work the following definition of an inverse H-bond in carbocations **I** and **II** can be deduced: it is a strong ionic bond formed by a hydrogen atom of a C–H^δ−^ bond with increased electron density and by the positively charged C^+^ atom of the carbocation. This bond can be regarded as resulting from the strong polarization of an olefin molecule by a carbocation and can be interpreted as an acid–base interaction. It was found that this type of interactions occurs in compounds formed by the same vinyl cations, C_3_H_7_^+^/C_4_H_9_^+^, with basic H_2_O and alcohols’ molecules^21^.

Thus, the ability of an H atom, depending on conditions, to be either an acceptor or a donor of electrons leads to the fact that the H-bonds it forms can be categorized as either direct (classic) or inverse ones.

## Methods

The salts of vinyl cations C_3_H_5_^+^ and C_4_H_7_^+^ with the CHB_11_Cl_11_^−^ anion and their crystals have been obtained and studied previously^15,16^. Acid H{Cl_11_} was synthesized according to the procedure described in Ref.^43^. Details of the obtaining of salts of cations C_3_H_5_^+^⋅C_3_H_6_ and C_4_H_7_^+^⋅C_4_H_8_ as well as crystals of salt C_4_H_7_^+^⋅C_4_H_8_{Cl_11_^−^} are given in section “Results”, because they are necessary to describe the results of the work. To obtain the salt under study, we used 1,2-dichloropropane and 1,4-dichlorobutane (both from Sigma-Aldrich, Inc., with 99% purity) without further purification. DCM as a solvent was dried by standard methods.

All sample handling was carried out in an atmosphere of argon (H_2_O, [O_2_] > 0.5 ppm) in a glove box. ATR IR spectra were recorded on a Shimadzu IRAffinity-1S spectrometer housed inside the glove box in the 4000–400 cm^−1^ frequency range using an ATR accessory with a diamond crystal. The spectra were processed in the GRAMMS/A1 (7.00) software from Thermo Scientific (Waltham, MA, USA).

All calculations were performed at the B3LYP^44^/6-311G++(d,p)^45^ level of theory with Grimme dispersion correction^46^, the HF and MP2 level theory with an ultrafine integration grid within the framework of the Gaussian 09 package^47^. All cations were calculated at singlet state. The vibrational frequencies were computed for all the studied structures, where the optimization converged successfully to the shallow local minima on the potential energy surface, as confirmed by the absence of negative/imaginary vibrational frequencies.

X-ray diffraction data were collected on a Bruker Kappa Apex II CCD diffractometer using φ,ω-scans of narrow (0.5°) frames with Mo Kα radiation (λ = 0.71073 Å) and a graphite monochromator at temperature 200 K. The structures were solved by direct methods with the help of SHELXT 2014/5 (Ref.^48^) and refined by a full-matrix least-squares anisotropic-isotropic (for H atoms) procedure using the SHELXL-2018/3 software suite^48^. Absorption corrections were applied by the empirical multiscan method in the SADABS software^49^. Positions of the hydrogen atoms for CH_3_ groups were calculated via the riding model. The hydrogen atom positions for C1A and C1B were located by means of a difference Fourier map. The crystallographic data and details of the refinements are given in Tables S1 and S2 in SI.

The resultant crystal structures were analyzed for molecular geometry and short contacts between non-bonded atoms using *MERCURY* programs^50^. CCDC 2309018 contain the supplementary crystallographic data for this paper. These data can be obtained free of charge via http://www.ccdc.cam.ac.uk/cgi-bin/catreq.cgi, or from the Cambridge Crystallographic Data Centre, 12 Union Road, Cambridge CB2 1EZ, UK; fax: (+ 44) 1223 336 033; or e-mail: deposit@ccdc.cam.ac.uk.

### Supplementary Information

Supplementary Information 1.

## Data Availability

The datasets used and/or analysed during the current study are available from the corresponding author on reasonable request.
