# Supplementary material for: A new type of C+⋯Hδ−(C=) bond in adducts of vinyl carbocations with alkenes
Source: Sci Rep. 2024 Apr 10;14:8423. doi: 10.1038/s41598-024-58109-4 (PMC11006867; doi:10.1038/s41598-024-58109-4)
Supplement: Supplementary file 1 — Supplementary Information 1. [file 41598_2024_58109_MOESM1_ESM.doc]

SUPPORTING INFORMATION

**A new type of C^+^‧ ‧ ‧ H^δ‒^(C=) bond in adducts of vinyl carbocations with alkenes**

*Evgenii S. Stoyanov ^*^, Irina Yu. Bagryanskaya and Irina V. Stoyanova*

N.N. Vorozhtsov Institute of Organic Chemistry, Siberian Branch of Russian Academy of Sciences, Lavrentiev Avenue 9, Novosibirsk 630090, Russian Federation.

* Corresponding author. E-mail address: [evgenii@nioch.nsc.ru](mailto:evgenii@nioch.nsc.ru)


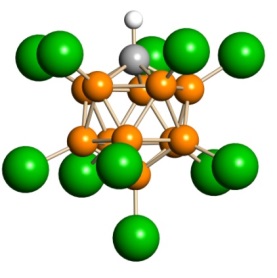


**Figure S1.** Icosahedral carborane anion, CHB_11_Cl_11_^−^, used in this work

3000

2500

2000

1500

2965

2933

2876

2892

3023

1532

1486

ν, cm^-1^

**Figure S2.** ATR IR spectra of the crystals of salt С_3_Н_5_^+^{Cl_11_^−^}, studied in ref. [16] (red) and solid grains of salt С_3_Н_5_^+^⸱C_3_H_6_{Cl_11_^−^}, which do not show X-ray diffraction patterns (blue). The spectra are normalized to the intensity of the CH stretch band of the {Cl_11_^−^} anion at 3023 cm^−1^.


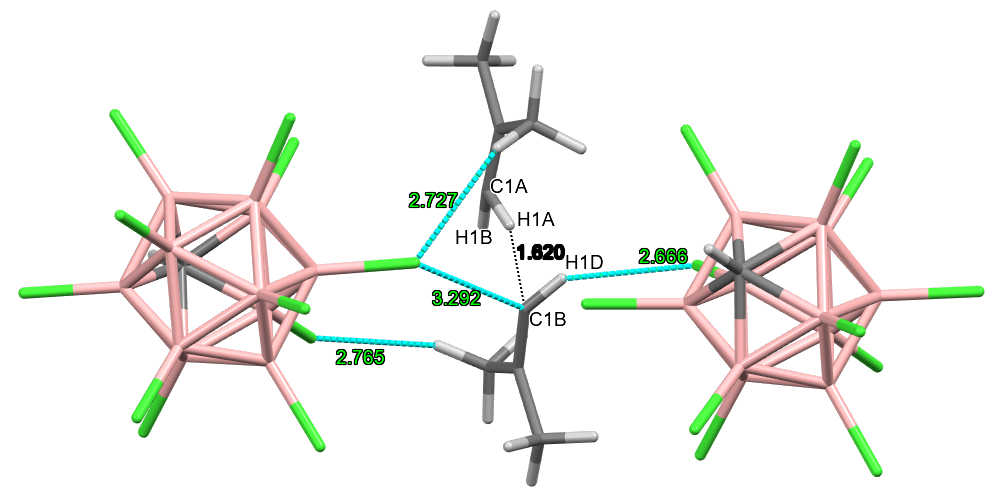


**Figure S3.** The shortest distances between C and H atoms (less than the sum of their van der Waals radii) of cationic adduct **I** (the isomer with 71% occupancy of the H1A atom) and Cl atoms of neighboring anions

**Table S1**

Crystal data and structure refinement for compound C_4_H_7_^+^ꞏC_4_H_8_(CHB_11_Cl_11_^−^)

| Compound | **SES22** |
| --- | --- |
| Empirical formula | C_8_H_15_ + B_11_Cl_11_CH |
| Formula weight | 633.08 |
| Temperature K | 200(2) |
| Wavelength Å | 0.71073 |
| Crystal system | Monoclinic |
| Space group | P2_1_/n |
| Unit cell dimensions *a* Å | 9.2299(11) |
| *b* Å | 18.186(2) |
| *c* Å | 15.655(1) |
| *β* ^o^ | 94.081(3) |
| Volume Å^3^ | 2621.1(5) |
| Z | 4 |
| Density (calcd.) Mg.m^–3^ | 1.604 |
| Abs. coefficient mm^–1^ | 1.167 |
| F(000) | 1248 |
| Crystal size mm^3^ | 0.06 x 0.10 x 0.20 |
| Θ range for data collection ° | 2.2 – 27.5 |
| Index ranges | -11 ≤ h ≤ 12, -23 ≤ k ≤ 23, -20 ≤ l ≤ 14 |
| Reflections collected | 23355 |
| Independent reflections | 6022 R(int) = 0.060 |
| Completeness to θ % | 99.9 |
| Data / restraints / parameters | 6022 / 7 / 293 |
| Goodness-of-fit on *F^2^* | 1.04 |
| Final R indices *I > 2σ(I)* | R_1_ = 0.0497, wR_2_ = 0.1414 |
| Final R indices (all data) | R_1_ = 0.0680, wR_2_ = 0.1570 |
| Largest diff. peak / hole e.Å^-3^ | 1.27/ -0.81 |
| CCDC |  |

Table S2. Selected bond lengths (Å) and bond angles (°) for cation **I** (two independent molecules).

| Cation A | | Cation B | |
| --- | --- | --- | --- |
| Bond | Length (Å) | Bond | Length (Å) |
| C1A–C2CA | 1.247(5) | C1B–C2CB | 1.250(5) |
| C2CA–C3CA | 1.460(7) | C2CB–C3CB | 1.462(6) |
| C2CA–C4CA | 1.465(7) | C2CB–C4CB | 1.459(8) |
| C1A–H1A | 0.87(4) | C1B–H1A’ | 0.89(8) |
| C1A…H1A’ | 1.664(4) | C1B…H1A | 1.620(8) |
| Bond angles | (°) | Bond angles | (°) |
| С1A–C2CA–C3CA | 121.8(4) | С1B–C2CB–C3CB | 121.7(4) |
| С1A–C2CA–C4CA | 117.3(4) | С1B–C2CB–C4CB | 117.2(4) |
| С1A–C2CA–C4CA | 121.0(4) | С1B–C2CB–C4CB | 121.2(4) |
| C2CA–C1A–H1A | 106(4) | C2CB–C1B–H1A’ | 109(8) |
| C1A– H1A–C1B | 155.4(4) | C1A– H1A’–C1B | 144.3(8) |

**Experimental details**

**Elemental analysis on C and H** was performed on automated CHNS Analyzer EURO EA 3000. The margin of error of the analytical results is 0.3 wt.% for neutral organic compounds containing a 100% molar proportion of the analyte. For the chloronium cation in the salt **II** the molar proportion of the analyte (C and H) is 13,7%, which significantly increases the analysis error. In addition, the counterion contains C and H and has exceptionally high thermal stability, which may lead to its incomplete atomization in the analyzer. Therefore, in order to reduce the error caused by the anion, a standard was used ⸻the crystalline salt *i*-C_4_H_9_^+^{Cl_11_^−^}⸻obtained as described in [15] (Table S2).

Results of determination of C (wt%) and H (wt%) in a sample of *i-*С_4_Н_9_^+^{Cl_11_^-^} crystals.

| Sample | sample weight, mg | C_4_, wt% | H_9_, wt% |
| --- | --- | --- | --- |
| Crystals of the salt  *i-*С_4_Н_7_^+^(CHB_11_Cl_11_^-^). | 1.102 | 12.35 | 2.47 |
| Chloronium salt  C_x+1_H_y+1_B_11_Cl_12_ | 1.659 | 12.36 | 2.37 |

Table S3. Chemical C/H analysis of cation C_x_H_y_^+^, in its salts with the {Cl_11_^-^} anion (salt **II**).

| Sample | sample weight, mg | C_x_, wt% | H_y_, wt% |
| --- | --- | --- | --- |
| The salt  С_x_Н_y_^+^(CHB_11_Cl_11_^-^) | 1.371 | 20.00 | 3.21 |
| The salt  C_x_H_y_^+^(CHB_11_Cl_11_^-^) | 1.446 | 19.32 | 3.09 |

Using the known composition of the salt *i-*С_4_Н_9_^+^{Cl_11_^-^} as a standard, the calculation of the composition of salt **II** gives the result С_6.25_Н_11.25_, which is close to the composition C_6_H_11_ or С_3_Н_5_^+^ꞏС_3_Н_6_.

**The details of quantum chemical calculations**

We optimized the structure of **I** at the UB3LYP/6-311++G(d,p) level of theory under three conditions:

1. Coordinates of atoms C1A, C1B, and H1A were fixed values determined by X-ray diffraction, whereas coordinates of all other atoms were optimized. In a structure optimized under these conditions, with a fixed ^+^C∙∙∙С distance of 2.437 Å (Figure S4), C=C bond length of the cation noticeably exceeds the experimental value (1.286 and 1.247 Å, respectively); the same is true for the butylene molecule (1.340 and 1.250 Å respectively). Such a considerable difference between the calculated and experimental values means that the optimized structure is unsatisfactory.


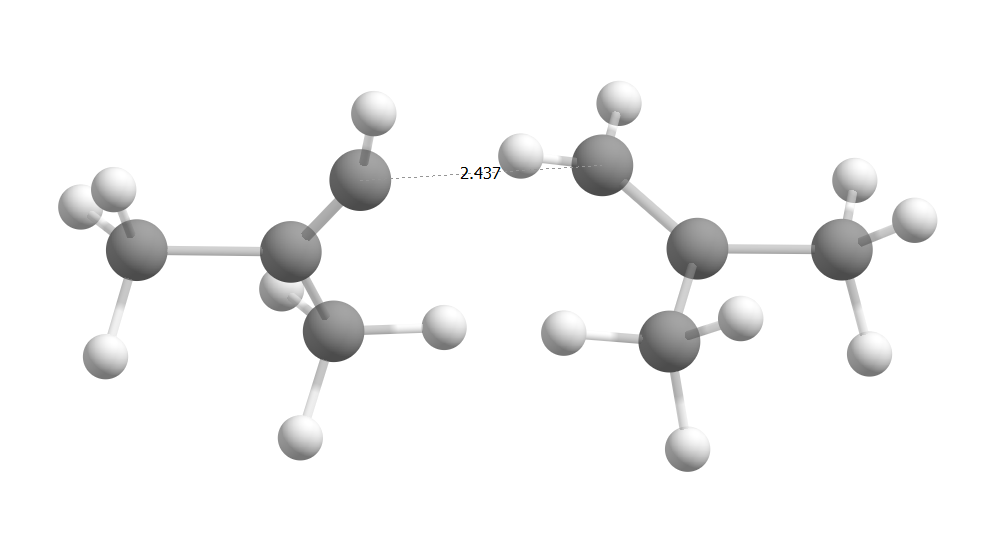


1.286

1.340

(*A*)

Figure S4. Calculated structure of cation **I**—with fixed C1A, C1B and H1A atoms of the C^+^···H^δ-^−C^δ+^ moiety—that matches the structure determined by X-ray diffraction. Optimized coordinates of all other atoms are presented too

2. The coordinates of all hydrogen atoms are optimized while the carbon atoms are fixed at coordinates determined by X-ray diffraction. Then, the central С^+^‧‧‧Н^‒^‧‧‧ С^+^ part of the structure becomes close to symmetrical (Figure S5) with a С‧‧‧С distance of 2.553 Å. It is much greater than the experimentally established value of 2.44 Å, i.e., it also contradicts experiment.


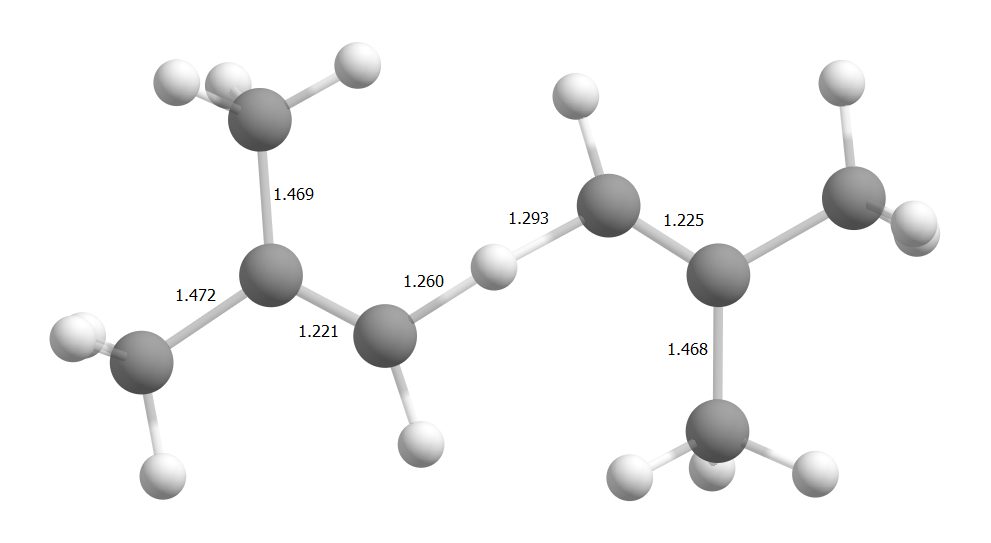


(*B*)

Figure S5. Calculated structure of cation **I** with optimized coordinates of all hydrogen atoms and fixed coordinates of the carbon atoms as determined by X-ray diffraction.

3. All atoms of adduct **I** were optimized. Then, both its C_4_H_7_ entities collapsed to one carbocation C_8_H_15_^+^ (*C*) with disappearance of the bridging H-atom.

The calculated energies of structures (*A*), (*B*), and (*C*) are 92.1, 76.3, and 0.0 kcal/mol, respectively. Thus, the energy state—of structure (*A*) of the adduct having a fixed localization of atoms of the C^+^‧‧‧H─C moiety—matching experimentally determined one is the most unfavorable. Additionally, the calculated IR spectra of (*A*) contain two intense imaginary bands with frequencies at −2142 and −1798 cm^-1^ belonging to δC_1B_-H_1A_ bending vibrations, and the νC_1B_-H_1A_ stretching vibration has an unacceptably high frequency at 5514 cm^−1^. In other words, the compound with structure (*A*), according to quantum chemical calculations, cannot exist.

The calculated energy of structure (*B*) with a symmetrical C^+^‧‧‧H^‒^‧‧‧C^+^ part of the molecule is also very high, and the calculated IR spectrum contains four imaginary frequencies at −319, −208, −148, and −121 cm^−1^, which involve vibrations of the C^+^‧‧‧H^‒^‧‧‧C^+^ group. All this indicates the impossibility of the existence of such a compound.

From the calculations, it follows that when the structure is optimized without any restrictions, then the two entities of adduct **I**, C_4_H_7_^+^ and C_4_H_8_, merge into one C_8_H_15_^+^ cation with a large energetic benefit. By contrast, this phenomenon was not observed experimentally. Therefore, calculations at the UB3LYP/6-311++G(d,p) level with Grimme dispersion correction,^37^ are not applicable to the research on vinyl carbocations. This conclusion has been reached earlier, in studies on all saturated and unsaturated non aromatic carbocations using a different quantum chemical calculation.^15,16,24,25^

In this study we also used calculations at the HF, and MP2^48^ level theory with an ultrafine integration grid within the framework of the Gaussian 09 package,^49^ and Minnesota functional MO62X. They yielded similar results. Therefore, further development of calculation methods as applied to carbocations is necessary with the participation of experienced quantum chemists in the investigation of this issue.
